# Intellectual disability content within tertiary medical curriculum: how is it taught and by whom?

**DOI:** 10.1186/s12909-018-1286-z

**Published:** 2018-08-02

**Authors:** Julian N. Trollor, Claire Eagleson, Beth Turner, Jane Tracy, Jennifer J. Torr, Seeta Durvasula, Teresa Iacono, Rachael C. Cvejic, Nicolas Lennox

**Affiliations:** 1 Department of Developmental Disability Neuropsychiatry (3DN), 34 Botany Street, UNSW, Sydney, NSW 2052 Australia; 2 Centre for Developmental Disability Health Victoria (CDDHV), Monash Health, 122 Thomas Street, Dandenong, VIC 3175 Australia; 3 Faculty of Medicine, Nursing and Health Sciences, Monash University, Clayton, VIC 3168 Australia; 4 Department of Psychiatry, Monash University, Monash Medical Centre, Block P, Level 3 246 Clayton Rd, Clayton, VIC 3168 Australia; 5 Centre for Disability Studies, Sydney Medical School, The University of Sydney, Level 1, Medical Foundation Building, 92-94 Parramatta Road, Camperdown, NSW 2050 Australia; 6 La Trobe Rural Health School, La Trobe University, 102 Arnold Street, Bendigo, VIC 3550 Australia; 7 Queensland Centre for Intellectual and Developmental Disability (QCIDD), Mater Research Institute, The University of Queensland, Level 2 Aubigny Place, Mater Hospitals, South Brisbane, QLD 4101 Australia

**Keywords:** Intellectual disability, Medical training, Medical education, Curriculum, Health inequalities, Teaching methods

## Abstract

**Background:**

Individuals with intellectual disability experience higher rates of physical and mental health conditions compared with the general population, yet have inequitable access to health care services. Improving the workplace capacity of medical professionals to meet the needs of this population is one way to reduce barriers to care and improve health outcomes. Using diverse pedagogy appropriate to learning outcomes to teach medical students about intellectual disability is a necessary step in improving future workplace capacity. However, there is a lack of research into how, and by whom, medical students are taught about intellectual disability. The aim of this study was to investigate this through an audit of Australian medical school curricula.

**Methods:**

The Deans of Australian universities that provide accredited medical degrees (*n* = 20) were invited by email to participate in a two-phase audit of intellectual disability content in the curricula. Phase 1 (*n* = 14 schools) involved the Dean’s delegate completing a telephone interview or questionnaire regarding medical course structure. If intellectual disability content was identified, a unit coordinator was invited to complete a survey regarding how this content was taught and by whom (Phase 2; *n* = 12 schools).

**Results:**

There was considerable variability across Australian medical schools regarding methods used to teach content about intellectual disability. Didactic teaching methods were most frequently used (62% of units included some form of lecture), but workshops and tutorials were reasonably well represented (34% of units contained one or both). Thirty-six percent of units included two or more teaching methods. Almost all schools (83%) used some problem- and/or enquiry-based learning. Educator backgrounds included medicine, nursing, and allied health. A majority of schools (*n* = 9, 75%) involved people with intellectual disability designing and teaching content, but the extent to which this occurred was inconsistent.

**Conclusions:**

Renewing curricula around intellectual disability across all medical schools by introducing varied teaching methods and the inclusion of people with intellectual disability would assist students to develop knowledge, skills, attitudes, and confidence in intellectual disability health. Such renewal offers the potential to reduce barriers to service this population regularly face, thereby improving their health outcomes.

## Background

### Unmet health needs of people with intellectual disability

Health service planning for people with intellectual disability requires special consideration due to the complexity of their health needs [1–3]. People with intellectual disability have a human right to equitable access to mainstream services. However at present, compared with the general population, people with intellectual disability encounter reduced access to preventive care, poor health promotion, significantly higher rates of undiagnosed disorders, inappropriate treatment [4–7], and early mortality from preventable causes [8, 9]. One of the foremost barriers to accessing health care services is that health professionals lack adequate training in intellectual disability [10–13].

The United Nations Convention on the Rights of Persons with Disabilities (UNCRPD) [14], and legislation such as the Disability Discrimination Act in Australia [15] compels governments and communities to make reasonable adjustments to practice or service provision to ensure people with disability have the same access to health services as the general population. In Australia, the National Disability Strategy [16] has set goals to achieve the best possible health outcomes for people with disability through improved health practitioner training. Mental health policy, such as the Living Well report [17] and the National Mental Health Workforce Plan [18] outline the need to include mental health content in university curricula to allow future health professionals to support people with intellectual disability, and people with complex health and social needs respectively. Despite the continued barriers to accessing services, there have been some positive developments. Several countries have introduced health assessment programs [19–22], and *The Guide* [23] and a set of core competencies [24] have been developed to encourage Australian mental health professionals to adopt a culture of accessibility. The roll-out of the National Disability Insurance Scheme (NDIS) within Australia, which began in June 2016, has provided a policy driver to change the way that disability and health services interact, improving treatment pathways and access [25]. Through the Information, Linkages and Capacity building arm of the NDIS (ILC; with the other arm providing NDIS plans for people with disability), the scheme aims to fund activities to build the capacity of mainstream and community services to be more inclusive of people with disability [26].

### The importance of intellectual disability in tertiary medical education

Medical professionals have reported that they lack the knowledge, skills and confidence to work with people with intellectual disability, and would like enhanced education [13, 27, 28]. Studies by members of our group indicate that training of future doctors in Australia lacks necessary intellectual disability health content to inform adjustments to practice [29, 30]. This suggests a compounding generational impact on future doctors unless action is taken to address the curriculum gap, with a lack of exposure to intellectual disability education associated with fewer graduates choosing to work in the area [31]. Piachaud [32] suggested that teaching core concepts, such as communicating effectively with people with intellectual disability, should begin in the first year of education, with exposure fostering positive attitudes [33]. Inclusive teaching, defined as individuals with intellectual disability developing or providing education, has also been found to positively influence students’ capability, attitudes and confidence in working with this population [34–37].

### Medical pedagogy and how content about intellectual disability is taught

Contemporary medical pedagogies (see Mann [38] for a summary) stress the importance of the learner as an active participant in his/her education, interaction with others to share knowledge, and learning while participating, all of which help students to critically evaluate information, make decisions, and solve problems [38–41]. The use of specific teaching methods depends on stated learning objectives. Lectures, a passive form of learning, are effective for conveying factual information, but less effective for promoting active thought and influencing attitudes [32, 42]. Skills are frequently taught using clinical rotations, high fidelity simulation (computerised manikins that simulate real-life scenarios), and interviews with standardised patients (someone portraying a patient with a standard set of symptoms) [43]. Medical curricula worldwide have evolved to include more problem-based learning (PBL) and enquiry-based learning (EBL), and summative and formative assessments [44–46]. A systematic review showed that PBL had a positive effect on graduate physicians’ social and cognitive skills and competencies, yet students tended to self-rate their resulting level of medical knowledge more negatively than those in a control group where more traditional didactic methods were utilised [47].

There have been only a few studies examining how intellectual disability is taught in medical schools. Lennox and Diggens [29] reported a comprehensive audit of Australian medical schools in 1999, and found considerable variation in how intellectual disability content was taught. Most compulsory units that addressed intellectual disability were taught using didactic lectures and seminars, and assessed using examinations. The elective units provided more opportunity than the compulsory units to develop skills and gain experience from people with intellectual disability, and assessments were more varied. A third of participating schools (*n* = 3) utilised inclusive teaching. Medical schools with an academic appointment in intellectual disability had, on average, a greater quantity of intellectual disability teaching than those without. Elsewhere, curricula in the United Kingdom (UK) with intellectual disability content and teaching methods such as actors with intellectual disability simulating patients [35, 48, 49] have been found to i) improve students’ ability to give accessible information to simulated patients, and gauge their understanding and ability to provide informed consent [48]; ii) detect previously undiagnosed disorders in community patients with intellectual disability [48]; and iii) choose suitable clinical approaches [49].

Examining disability research more broadly, an earlier but comprehensive survey of disability teaching across UK medical schools in 1994 showed that all 23 schools used lectures and seminars, while 15 included visits to disability and rehabilitation centres [34]. Videos were frequently used, but rarely role-play. Some schools used innovative techniques, such as a drama program and seminars run by people with intellectual disability. In another example of a curriculum to teach medical students about disability, Symons et al. [50] found that the State University of New York curriculum included didactic teaching, meetings with simulated patients, clinical experience in local primary care services, meeting individuals with disability and their families in the community, presentations by patient advocates, and visits to community disability agencies. Symons et al. [50] suggested that including the program early in the students’ education improved their perception that providing services to people with disability is a natural and expected part of general patient care.

### Medical program accreditation policy and standards

The focus of this study was tertiary curricula in Australian medical schools. In this context, the Australian Medical Council (AMC) is responsible for developing accreditation policy, and accrediting courses against a set of standards [51]. Each university develops their own curriculum that will allow graduates to meet the AMC’s graduate framework of outcome statements (indicating that graduates are ready to practice clinically). The framework evolved from policy in Australia moving towards competency-based education [52]. While the AMC standards do not refer to intellectual disability specifically, they include statements that medical education should include a range of learning and teaching methods including working with patients under supervision, appropriate assessments to evaluate learning objectives, and that students should learn from and about other health professionals. The standards do not state specifically who should teach the content, nor how often the curriculum should be reviewed (only stating that it should be regularly monitored). The AMC Medical School Accreditation Committee advises on national and international developments concerning medical education, and encourages and undertakes endeavours to improve medical education [53].

### The current study

There has been no examination of how intellectual disability health content is taught in Australian medical schools since that of Lennox and Diggens’ audit [29] published over 15 years ago. Thus, an up-to-date audit is required for researchers and educators to identify if and where there are gaps in teaching (which may be contributing to poor workforce capacity), and identify areas for improvement that could be targeted with education enhancement strategies. An audit would also provide a baseline to assess the effects of any such strategy. In response, our team conducted an audit of curricula across Australian medical schools. *What* is taught was reported in Trollor et al. [30], while the aim of the current study is to quantify and describe *how* intellectual disability content is taught, and by whom. Regarding how content is taught, the audit aimed specifically to assess i) the methods and pedagogies employed to present intellectual disability content to help determine whether they are sufficient to teach students the necessary knowledge, skills and attitudes around intellectual disability; and ii) whether intellectual disability content was assessed, considering the importance of assessments for the retention of information [54]. Regarding who taught the content, the audit aimed specifically to examine i) the professional background and appointment type educators held to assess if students are learning from a range of perspectives; and ii) how many staff had expertise or an interest in intellectual disability to estimate how many educators could potentially develop and promote intellectual disability education across their institution, and act as collaborators in any future projects aimed at enhancing education in this area. We hypothesised that since Lennox and Diggens’ [29] audit, there is more consistency in how intellectual disability is taught, especially with the release of additional guidelines and legislation in the intervening years (e.g. the National Disability Strategy [16]). This audit represents the first step in determining the need for a multi-phase strategy proposed by the Department of Developmental Disability Neuropsychiatry, UNSW Sydney to renew medical intellectual disability curricula through the creation of a toolkit for universities.

## Methods

### Recruitment and materials

An audit of Australian medical school curricula examining intellectual disability content was conducted in two phases from 2013 to 2014. The recruitment and data collection process, and materials used have been described in detail in Trollor et al. [30]. In brief, Deans of the 20 medical schools that offer AMC accredited medical degrees were invited to participate in the audit via email. Fourteen medical schools agreed to participate in Phase 1, which involved an interview about the structure of the medical course. Intellectual disability content was identified in the curricula of 12 schools, with course coordinators then participating in Phase 2. The course coordinators from the 12 participating medical schools completed a staff survey designed to explore the methods used to teach intellectual disability content and the professional backgrounds of those who taught it. The results of the current study were based on the responses from these 12 schools (60% response rate). See Trollor et al. [30] for a full list of questions used in the interview and survey. Descriptive analysis was used to examine the results.

### Nature of medical degree courses

Information about the nature of medical degree courses included in the audit has been published previously [30]. In brief, a median of 182 students were enrolled across each medical degree course (range = 99–520). The 12 participating medical schools provided a total of 42 compulsory units in which intellectual disability was taught, referred to as intellectual disability units (range = 1–12 compulsory units per school; median = 2). Intellectual disability units denote discrete course components containing some auditable content specific to intellectual disability. Teaching time focused on intellectual disability content per compulsory unit ranged from 30 min to 18 h (median = 2.55 h). Six participating medical schools provided eight elective intellectual disability units (range = 1–3 elective units per school; median = 1). The time spent teaching intellectual disability content per elective unit varied from 1 h to 222 h (median = 3 h). Only a small proportion of students completed the elective units (median = 12.5; range = 3–180 students per unit).

## Results

### Teaching method and assessment

Intellectual disability units were taught using a range of teaching methods (see Table 1). For compulsory units, the most frequent method was lectures (67% of compulsory units included some form of lecture), and the least frequent were other methods, such as a clinical assessment and a clinical day (each 2%). In 15 compulsory units, content was delivered using two or more teaching methods (36%). Six schools used at least two different teaching methods across their compulsory units. For elective units, the methods used were reasonably evenly represented. In three elective units, two or more teaching methods were used (38%). The majority of compulsory units (33 units; 79%) and elective units (five units; 63%) had intellectual disability content in the examinations or other forms of assessment. Two schools did not have any compulsory units in which intellectual disability content was assessed by examinations or assignments.

**Table 1:** Teaching method used for intellectual disability content

	Compulsory Units *N* = 42	No. schools across which compulsory units taught *N* = 12^+^	Elective Units *N* = 8	No. schools across which elective units taught *N* = 6	Total Units* *N* = 50	No. schools across which units taught *N* = 12
Lecture	28	8	3	3	31	9
Workshop	8	4	4	3	12	5
Other	9	3	3	3	12	5
*Case-based*	*7*	*2*	*0*	*0*	*7*	*2*
*Clinical assessment*	*1*	*1*	*0*	*0*	*1*	*1*
*Clinical coaching and practicals*	*0*	*0*	*1*	*1*	*1*	*1*
*Clinical placement*	*0*	*0*	*1*	*1*	*1*	*1*
*Clinical day*	*1*	*1*	*0*	*0*	*1*	*1*
*Self-directed learning*	*0*	*0*	*1*	*1*	*1*	*1*
Tutorial	5	3	2	1	7	3
No method listed^	7	4	0	0	7	4

^+^Results from the 12 schools surveyed (out of 20 approached; 60% response rate)

*Total units = compulsory and elective units combined

^Some units based in inpatient or community/mental health setting

### Pedagogy

PBL or EBL was utilised for the majority of compulsory units to teach intellectual disability content (57%), and in two units a combination of these pedagogies were used (5%, see Table 2). The only pedagogy used for elective units was EBL (four units; 50%). Pedagogy for units taught by two schools was unknown.

**Table 2:** Pedagogy used for intellectual disability content

Pedagogy	Compulsory Units *N* = 42	No. schools across which compulsory units taught *N* = 12	Elective Units *N* = 8	No. schools across which elective units taught *N* = 6	Total Units* *N* = 50	No. schools across which units taught^ *N* = 12
Problem-based only	16	5	0	0	16	5
Enquiry-based only	8	5	4	3	12	5
Combination	2	2	0	0	2	2
No pedagogy listed	16	7	4	4	20	8

*Total units = compulsory and elective units combined

^Pedagogy unknown for all the units taught in 2 schools

### Staff appointment

Each staff member who taught intellectual disability units had one of three appointment types: i) an ongoing or fixed university appointment (university); ii) a position external to the university, that is a guest lecturer/speaker (external); or iii) a conjoint position with the university, that is an honorary teaching appointment held by medical, allied health and other professionals (conjoint). As shown in Table 3, it was most common for compulsory intellectual disability units to be taught exclusively by staff with university appointments (12 units; 29%), followed by a combination of university and externally appointed staff (nine units; 21%). Elective intellectual disability units were most frequently taught by a combination of staff with university or external appointments (three units; 38%). Overall, 40% of units (compulsory and elective combined) were taught by multiple staff who each held different appointment types.

**Table 3:** Staff appointments for teaching intellectual disability content

Appointment	Compulsory Units *N* = 42	No. schools across which compulsory units taught *N* = 12	Elective Units *N* = 8	No. schools across which elective units taught *N* = 6	Total Units* *N* = 50	No. schools across which units taught^ *N* = 12
University only	12	6	2	2	14	7
External only	2	2	2	2	4	3
Conjoint only	3	3	1	1	4	4
University/External	9	7	3	1	12	7
University/Conjoint	4	1	0	0	4	1
External/Conjoint	1	1	0	0	1	1
University/External/Conjoint	3	2	0	0	3	2
Staff appointment unknown	8	1	0	0	8	1

*Total units = compulsory and elective units combined

^Staff appointment unknown for all units taught in 1 school

### Staff professional background

Details on staff background are provided in Table 4. Medical practitioners with general practitioner (GP), paediatric or other non-psychiatric medical backgrounds were involved in teaching most compulsory (62%) and elective (75%) intellectual disability units. The least frequent health background for compulsory units was registered nursing (2%), and for elective units it was psychology and registered nursing (both 25%). Thirteen compulsory (31%) and four elective units (50%) were taught by multiple staff from different backgrounds. Half of all units were taught exclusively by medical professionals (GP, paediatric, other medical backgrounds and psychiatric). As previously published in Trollor et al. [30], people with intellectual disability were involved in the delivery of intellectual disability content as tutors, lecturers or as symposium panel members for 24% of all units. This figure included nine compulsory units (across seven schools) and three elective units (across three schools).

**Table 4:** Professional background of staff teaching intellectual disability content

Profession	Compulsory Units *N* = 42	No. schools across which compulsory units taught *N* = 12	Elective Units *N* = 8	No. schools across which elective units taught *N* = 6	Total Units* *N* = 50	No. schools across which units taught *N* = 12
Medical Practitioner, non-psychiatrist	26	9	6	4	32	10
Medical Practitioner, psychiatrist	10	7	3	2	13	7
Allied health	6	4	5	3	11	6
Psychologist	5	3	2	2	7	4
Non-health background	3	3	0	0	3	3
Registered nurse	1	1	2	1	3	2
Staff professional background unknown	8	1	0	0	8	1
Person with intellectual disability involvement	9	7	3	3	12	9

*Total units = compulsory and elective units combined

### Staff interest and expertise

Nine schools had staff members who specialised or had a particular interest in intellectual disability and these schools offered an average of 4.67 intellectual disability units (compulsory and elective; range = 1–12 units). The three schools without staff members who specialised or had a particular interest in intellectual disability offered an average of 2.67 intellectual disability units (range = 2–3 units).

## Discussion

The way in which intellectual disability units were taught and who taught them varied markedly across medical schools. Lectures remain the most frequent teaching method used, despite a historical trend for a reduction in lecture-based teaching in Australian medical schools [55]. Nonetheless, there was a fair representation of practical and interactive teaching methods across some schools, perhaps as a result of recent literature about changing pedagogies in medical teaching [39–41] and the global trend towards student-directed learning and teaching in medical school courses [45, 46]. Although educators with a medical background taught the most units, education was also provided by professionals from other health backgrounds, providing at least a proportion of students with knowledge on intellectual disability from varied health perspectives. However, not all students had the opportunity to learn about intellectual disability using varied or innovative methods. Our previous findings indicated very limited intellectual disability health content in medical curricula [30]. Compounding this, the limited range of teaching methods used in some schools suggest that a considerable proportion of students will likely miss out on the opportunity to develop, in particular, the skills and attitudes required to address the considerable health needs of people with intellectual disability [1–3].

### Comparison with past research

Findings from the current audit largely reflect those found by Lennox and Diggens [29] in that there was wide variation across universities in the teaching methods used. This is likely in part due to AMC policy that all medical schools develop their own curricula [51]. Lennox and Diggens [29] found that didactic teaching, largely in the form of lectures, was most frequently utilised (34% of teaching hours), and that elective units offered a greater opportunity to develop practical skills compared with compulsory units (although only a small percentage of students were enrolled in them). These findings were reflected in the current study. Of note was a rise in the percentage of compulsory units that incorporated teaching strategies that were inclusive of people with intellectual disability (30% of schools in Lennox and Diggens [29]). Increased awareness of the rights of people with disability as championed in the UNCRPD [14] provides impetus for government and the community to engage in practices that work towards the inclusion of people with disability in community life and ending discrimination. Such impetus may be influencing greater inclusive teaching practices.

The percentage of units that contained some assessment of intellectual disability content appears to have decreased since Lennox and Diggens’ [29] audit (they found 89% of compulsory and 100% of elective units assessed intellectual disability content). As students will focus their attention on learning information that is required for assessments, the inclusion of assessment of content focused on the health and health care of people with intellectual disability is critical to student motivation and engagement, and is an important part of successful learning [54]. The percentage of all units taught exclusively by medical professionals was found to have increased from 36% reported by Lennox and Diggens [29]. In both audits, however, it was evident that individuals with a range of professional backgrounds were involved in teaching.

Kahtan et al. [34] also found considerable inconsistency in how disability was taught in UK medical schools. Teaching opportunities, such as clinic days and the participation of people with intellectual disability in symposia and tutorials were provided in some units audited in the current study. Still, no Australian medical schools appear to approach the breadth of intellectual disability content covered by curricula in the UK [35, 48, 49], and the broader disability curriculum employed at the State University of New York [50].

### Audit findings in relation to medical education research and policy

The finding in the current audit that lectures continue to be used frequently may reflect their perceived role in providing students with factual information [32], although there is evidence against their use for other aspects of learning, such as shaping attitudes [42]. In contrast, diverse methods of delivery, such as workshops, clinic visits, and placements are more likely to enhance students’ ability to critically evaluate information in order to make decisions, develop new solutions, and modify attitudes when working with people with intellectual disability [32, 38, 42]. Lectures also require fewer teaching staff and resources than other methods such as workshops, which is likely another reason they continue to be used frequently. Case-based learning, which was employed by two schools, involves students working through patient cases. This method may be useful for students to comprehend the complex health needs that some people with intellectual disability experience, the patterns of behaviour associated with certain genetic disorders that cause intellectual disability (behavioural phenotypes), and to consider reasonable adaptations to practice [23]. Exposure to different disability services and health providers during clinic visits and placements can also improve graduates’ ability to collaborate in multidisciplinary environments and their knowledge of care pathways [56]. In the current study, just over half of units that incorporated PBL/EBL also included lectures to teach intellectual disability content. This may be the preferred scenario for students as they can feel confident they are learning core intellectual disability content, and then build on this knowledge through learner-directed pedagogies [47], aiding in the development of critical thinking and problem solving skills through active participation and collaboration with other students [38–41].

With regards to who taught intellectual disability content, for some units the variety of staff appointments (university, external and conjoint) and professional backgrounds may have exposed students to varied viewpoints, skills, and practices. People with intellectual disability can have diverse physical and mental health needs, necessitating a multidisciplinary approach often across multiple sectors (e.g. health, disability, advocacy, education, employment, housing, transport, and justice). Contact with individuals from varied professional backgrounds during university could assist medical students to be aware of the roles and responsibilities of different professions with regards to intellectual disability health and wellbeing, services that are available, who can be contacted for referrals, and encourage cross sector collaboration [23]. It also fulfils AMC accreditation standards that state that students should learn from a variety of health professionals [51]. Schools that had staff with a specialisation or interest in intellectual disability offered more intellectual disability units than those without, a finding that is consistent with previous research [29]. Greater representation of educators with experience or an active interest in intellectual disability would likely have a positive impact on intellectual disability content in the curriculum, and would allow for the provision of appropriate support to students wishing to pursue a future career in the area. Medical practitioners working and teaching in the area can act as role models and mentors for interested medical students.

Curricula can also be greatly bolstered by inclusive teaching [48]. In addition to being one way to meet human rights legislation requirements [14, 15], inclusive teaching practice provides a powerful approach for changing attitudes and perceptions [35, 36]. Such practice also ensures that students learn how they can successfully adapt their practice from individuals who receive these services [34, 36, 37, 48, 49]. Graduates who successfully learn how to adjust practice when working with people with intellectual disability through diverse educational exposure will likely be in a stronger position to contribute to meeting Australia’s obligation to provide equitable services for those with intellectual disability as outlined in human rights [14] and anti-discrimination legislation [15], and health policy [16–18]. It may also address the NDIS ILC objectives of strengthening the capacity of mainstream systems to meet the needs of people with disability [26]. Despite some changes apparent from the results of the current audit in comparison with those of Lennox and Diggens [29], it was evident that many students at graduation still will not have received such education.

### Limitations and directions for future research

This audit has limitations and the results should be interpreted with caution. Ascertainment bias may have influenced the results in that medical schools with intellectual disability content may have been more likely to take part. As data were collected remotely by self-report questionnaire, there may have been inflation in the reporting of the amount of intellectual disability teaching in the curricula, or, conversely, some details about the way the content was taught may not have been included. Further, as some questions were open-ended, they may have been interpreted differently by respondents. Forced choice questions may not have offered all possible response options. Finally, there were inconsistencies across medical schools regarding their course structure, and variable definitions of units of study that made direct comparisons of curricula across schools difficult. For future research, more details could be gathered about how people with intellectual disability were involved in teaching, and the nature of other teaching methods that were described (such as clinic days). Future audit surveys could also include items about whether other common teaching methods, such as inter-professional teaching, E-learning, or simulation (which no respondents mentioned) are utilised.

### Educational implications

In light of the current findings, we urge medical schools to evaluate how they teach medical students about intellectual disability health, and take steps to incorporate varied methods that encourage self-directed learning, practical experience, and inclusive teaching [32, 34–37]. There is also a need for regulatory and accreditation organisations to advocate for more education in this area [57]. The authors aim to develop, evaluate and implement a national education framework toolkit for medical school programs with the goal of renewing curricula in intellectual disability physical and mental health. The framework would support medical students to develop the knowledge, skills, confidence, and attitudes to successfully work with people with intellectual disability by providing up to date, evidence-based teaching materials and methods to be incorporated into existing curricula. Areas requiring improvement include the greater involvement of people with intellectual disability in the teaching process, increased frequency of assessment of intellectual disability content, and ensuring that teaching methods are sufficiently diverse to accommodate different learning outcomes, rather than relying so heavily on didactic lecture formats.

There are competing demands for curriculum space in medical degree courses. To augment intellectual disability health teaching, educators could be encouraged to see that education in this area can also have significance for wider population health and policy issues. Students can learn skills and knowledge that are not only relevant for caring for people with intellectual disability, but also for other populations, especially those that are disadvantaged or have complex needs. This can include the management of chronic, complex or rare health conditions; preventative health in vulnerable or ‘at risk’ population groups; alternative and augmentative communication methods; and law, ethics and human rights issues [57].

## Conclusions

The complex health needs of people with intellectual disability, their inequitable access to health care and poor health outcomes [2, 3] provide a strong rationale for medical graduates to gain a better understanding of this population’s health needs and how to meet these needs. As members of society, people with intellectual disability have a right to equitable access to mainstream health care and to health care that is specific to their disability (Article 25 of UNCRPD) [14]. Given that health care is delivered by mainstream services, with some exceptions [57], all practicing medical graduates will provide clinical care to people with intellectual disability [29].

For students from approximately half of the schools audited, there appears to be a relatively sound balance of teaching and learning methods that would assist students to learn not only knowledge, but also skills and attitudes required to work with people with intellectual disability. However, a considerable number of students are still receiving education via limited methods. The fall in assessment of intellectual disability content is of concern as it suggests information learnt may not be retained over the long term. A fair proportion of students look to be learning content from individuals with intellectual disability and staff with diverse professional backgrounds and appointment types, providing a breadth of knowledge around the complex physical and mental health needs of this population. It was a constructive finding that a majority of schools employed a staff member with expertise or an interest in intellectual disability as these individuals could potentially collaborate on future projects aimed at enhancing intellectual disability content in medical curricula. Enhanced training throughout medical school education is an important part of equipping health professionals with appropriate attitudes and knowledge to impact health outcomes for this group. It is recommended that Australian medical schools respond by reviewing their curricula to ensure that medical students receive comprehensive and varied education in this area.

## Abbreviations

AMC: Australian Medical Council
EBL: Enquiry-based learning
ILC: Information, Linkages and Capacity building
NDIS: National Disability Insurance Scheme
PBL: Problem-based learning
UK: United Kingdom
UNCRPD: United Nations Convention on the Rights of Persons with Disabilities
